# Long-term efficacy of Waveflex semi-rigid-dynamic-internal-fixation system in delaying intervertebral disc degeneration at adjacent segments and improving spinal sagittal imbalance

**DOI:** 10.1038/s41598-024-60940-8

**Published:** 2024-05-07

**Authors:** Wenxin Gao, Xiaoying Wang, Yungang Chen, Guoyan Liu, Pengfei Hou, Cunliang Guo, Xu Yang, Yanke Hao

**Affiliations:** 1 The First Affiliated Hospital, Zhejiang Chinese Medical University, Hangzhou, Zhejiang China; 2 Jinan Vocational College of Nursing, Jinan, Shandon China; 3 Shandong University of Traditional Chinese Medicine, Jinan, Shandong China; 4 Affiliated Hospital of Shandong University of Traditional Chinese Medicine, 16369 Jingshi Road, Jinan, Shandong China; 5 Tianjin University of Traditional Chinese Medicine, Tianjin, China; 6 Qingzhou People’s Hospital, Weifang, Shandong China

**Keywords:** Waveflex semi-rigid dynamic fixation system, Lumbar degenerative disease, Adjacent segmental degeneration, Imaging parameters, Musculoskeletal system, Bone

## Abstract

The Waveflex semi-rigid-dynamic-internal-fixation system shows good short-term effects in the treatment of lumbar degenerative diseases, but there are few long-term follow-up studies, especially for recovery of sagittal balance. Fifty patients with lumbar degenerative diseases treated from January 2016 to October 2017 were retrospectively analysed: 25 patients treated with Waveflex semi-rigid-dynamic-internal-fixation system (Waveflex group) and 25 patients treated with double-segment PLIF (PLIF group). Clinical efficacy was evaluated by Visual Analogue Scale (VAS) and Oswestry Disability Index (ODI). Imaging data before surgery and at 3 months, 1 year, and 5 years postoperatively was used for imaging indicator assessment. Local disc degeneration of the cephalic adjacent segment (including disc height index (DHI), intervertebral foramen height (IFH), and range of motion (ROM)) and overall spinal motor function (including lumbar lordosis (LL), pelvic incidence (PI), sacral slope (SS), pelvic tilt (PT), and |PI-LL|) were analysed. Regarding clinical efficacy, comparison of VAS and ODI scores between the Waveflex and PLIF groups showed no significant preoperative or postoperative differences. The comparison of the objective imaging indicators showed no significant differences in the DHI, IFH, LL, |PI-LL|, and SS values between the Waveflex and PLIF groups preoperatively and 3 months postoperatively (*P* > 0.05). These values were significantly different at 1 and 5 years postoperatively (*P* < 0.05), and the Waveflex group showed better ROM values than those of the PLIF group (*P* < 0.05). PI values were not significantly different between the groups, but PT showed a significant improvement in the Waveflex group 5 years postoperatively (*P* < 0.05). The Waveflex semi-rigid dynamic fixation system can effectively reduce the probability of intervertebral disc degeneration in upper adjacent segments. Simultaneously, patients in the Waveflex group showed postoperative improvements in LL, spinal sagittal imbalance, and quality of life.

## Introduction

Within natural aging and lumbar spine degeneration, lumbar degenerative disease is characterized by hypertrophy of facet joint and ligamentum flavum, degeneration of intervertebral disc, collapse of intervertebral space, and osteophyte formation^1,2^. This disease can cause pain and disability, place a heavy burden on the health care system, and restrict economic development. The global incidence of low back pain caused by lumbar degenerative diseases is estimated at 3.6%, compared with 4.5% in North America^3^. Furthermore, in 2010, disability caused by low back pain was reported to have seriously impaired the quality of life of 83 million patients^4^. Recently, the development of medical industry has increased diagnosis and treatment efficacy of lumbar degenerative diseases; however, with the aging of the population, disease burden, including disability and costs, is also increasing. Lumbar fusion has always been regarded as the gold standard for treatment of serious lumbar degenerative diseases. Traditional posterior lumbar interbody fusion (PLIF) and transforaminal lumbar interbody fusion are the most widely used surgical approaches^5,6^. However, after rigid fixation and fusion surgery, the movement of the repaired spinal segment is lost, and rigid fixation will subject the whole spine to abnormal biomechanical forces, leading to symptomatic degenerative changes in the adjacent active segments^7^. Simultaneously, rigid fixation and fusion have been shown to affect the range of motion (ROM) of the lumbar spine, affecting movements such as bending or squatting^8^. Therefore, procedures allowing for maintenance of a certain lumbar spine ROM while relieving pain and numbness have become a hot topic in recent clinical research.

Dynamic internal fixation aims at delaying adjacent segmental degenerative diseases and has shown good improvements in neurogenic lower back and leg pain, preserving a certain lumbar vertebrae ROM, and improving surgery safety and quality of life of patients^9^. A biomechanical study showed that, by preserving spinal physiological functions, the dynamic internal fixation system can minimize the pressure at and near the spinal instrumentation level^10^. Furthermore, it maintains the intervertebral disc's structure and function by controlling the segmental motion to achieve a relatively stable spinal state, while also reducing the risk of accelerated degeneration of the adjacent intervertebral discs^11^. The Waveflex semi-rigid-dynamic-internal-fixation system includes a pedicle screw dynamic internal fixation system, which combines traditional rigid fixation with modern elastic dynamic fixation. Under the premise of preserving partial motion of the instrumented segment, elastic dynamic fixation uses a pre-bent rod with two or three folds of undulating elastic structure, which can effectively limit the excessive flexion and extension range of the spine, effectively disperse the stress concentrated at the intervertebral disc of the adjacent segments, and delay its degenerative changes. Rigid fixation can maintain the intervertebral space height during operation, help dispersing the exercise load at the posterior facet joint and intervertebral disc, and effectively reduce facet joint pain incidence^12^. Although the Waveflex semi-rigid-dynamic-internal-fixation system has been shown to have good short-term efficacy and high safety for the treatment of a variety of lumbar degenerative diseases^13^, there is a lack of long-term follow-up studies providing reliable evidence for clinical and surgical decisions.

Sagittal spinopelvic alignment (SSPA) is a major determinant of health-related quality of life and is widely used to monitor the progression of compensatory mechanisms^14^. Changes in SSPA play a key role in pain and disability in patients with spinal degenerative diseases^15^. By coordinating the relationship between the spine, pelvis, and lower limbs during walking, especially in the sagittal plane, people maintain a stable upright posture consistent with human biomechanics^16^. Pathological changes of any segment of the trunk or lower limbs of patients with degenerative musculoskeletal disease can lead to overall posture balance modifications, leading to compensatory changes in other segments^17^.

Although the improvement of SSPA parameters has always been an options to evaluate surgical quality and clinical efficacy, no research has analysed whether the Waveflex semi-rigid-dynamic-internal-fixation system can effectively improve the sagittal imbalance of patients. Concomitantly, most studies focus on the evolution of local disc degeneration after surgery. However, there is a lack of studies evaluating the overall motor function of the spine. This study aimed analyse the long-term clinical effects and imaging parameters of the Waveflex semi-rigid-dynamic-internal-fixation system on patients with lumbar degenerative diseases, in order to provide detailed and reliable data to support clinical treatment and scientific research.

## Materials and methods

### Ethical approval

Ethical approval for this study (2023-LS-Number34-KY) was provided by the Affiliated Hospital of Shandong University of Traditional Chinese Medicine's institutional research ethics committee, Jinan, Shandong, China on 12 March 2023, and written informed consent was obtained from all patients. Written informed consent was obtained from all included patients, and the study was conducted in accordance with the Declaration of Helsinki and the STROBE criteria.

### Study design

**Figure 1:**
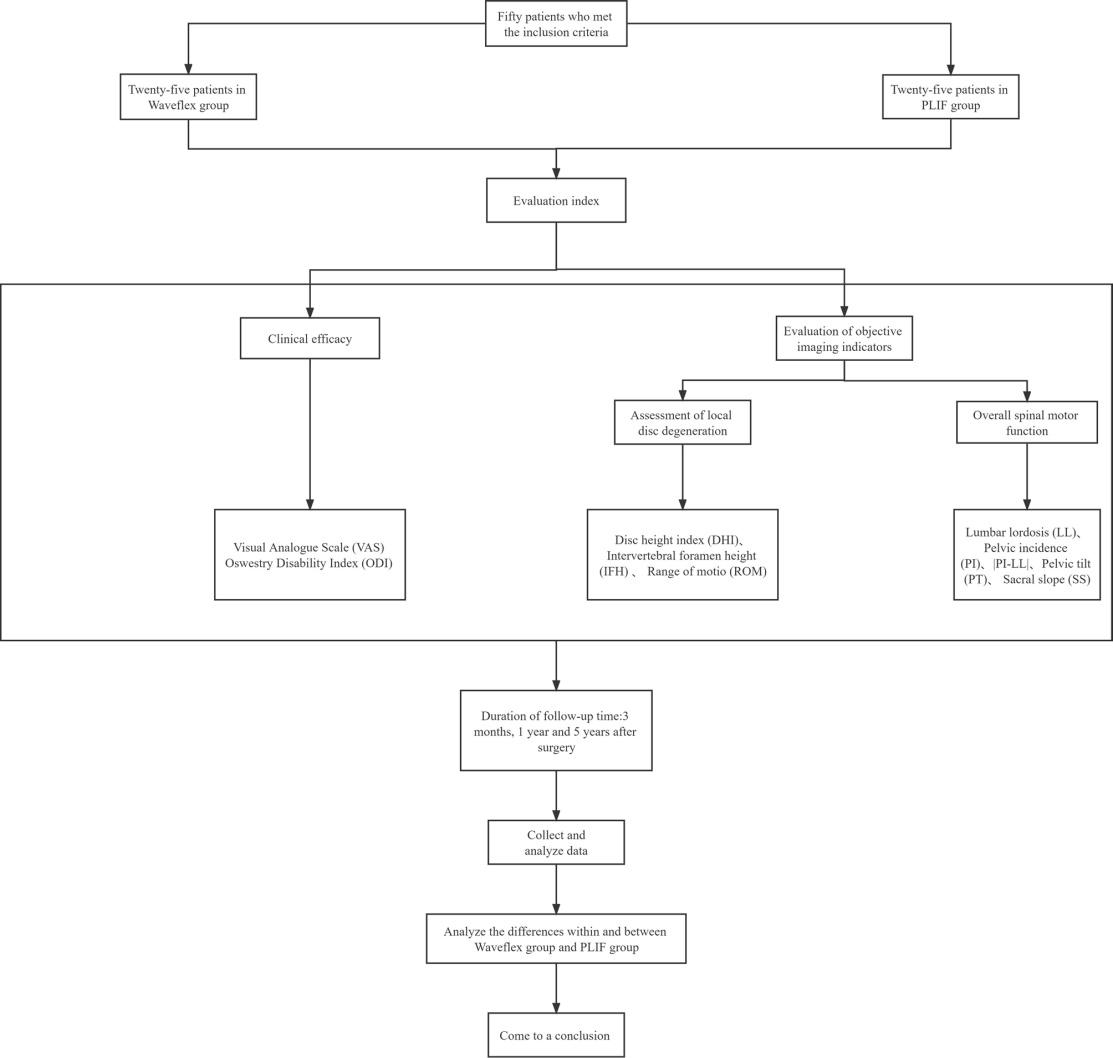
Study flowchart.

This study included 50 patients with lumbar degenerative diseases who were hospitalized and operated in the Department of Spinal Orthopaedics, the Affiliated Hospital of Shandong University of traditional Chinese Medicine from January 2016 to October 2017, including 25 patients treated with the Waveflex semi-rigid internal fixation system (Waveflex group) and 25 patients treated with double-segment PLIF (PLIF group). All patients were strictly screened according to the inclusion and exclusion criteria. All medical and imaging records were collected according to the inclusion criteria and were followed up. The study flowchart is shown in Fig. 1.

### Waveflex semi-rigid-dynamic-internal-fixation system

The new internal fixation system, made by Medyssey Company from South Korea, consists of three parts: titanium rod, screw, screw plug. Waveflex titanium rod: The titanium alloy Wave structure is both rigid and elastic, retaining a certain range of motion of the fixed segment (flexion 10°, extension 5°), the rod end is flat, increasing the contact surface between the screw and the rod, and the fixation is reliable. Screw: low notch with hydroxyapatite coating to enhance bone ingrowth; Screw plug: rectangular thread, not easy to misbuckle, slip wire.

### Inclusion criteria

The inclusion criteria were the following: (1) patients with double-segmental lumbar degenerative diseases (lumbar disc herniation, lumbar spinal stenosis, lumbar spondylolisthesis) with typical symptoms of lower back pain and nerve root involvement; consistent clinical symptoms, signs, and imaging data, and clear diagnosis; (2) no improvements for at least half a year following conservative treatment, requiring surgical treatment; (3) patients treated with Waveflex semi-rigid internal fixation (Medyssey company, Korea) or those who received double-segment PLIF; and (4) patients with complete data regarding clinical function scores and imaging results data.

### Exclusion criteria

Patients were excluded according to the following exclusion criteria: (1) patients with preoperative severe degeneration of superior adjacent segments, such as obvious loss of height, definite instability, or an intervertebral disc degeneration Pfirrmann grade > 2^18^; (2) patients with other lumbar diseases, such as lumbar infection, tumour, severe scoliosis, or cervical or thoracic spinal cord injury; (3) patients with incomplete follow-up data or with a follow-up period shorted than 60 months.

### Surgical scheme selection criteria

In the Waveflex group, the comprehensive analysis of symptoms, signs and imaging showed that the fusion segment was the segment mainly responsible for degeneration, requiring complete decompression, fixation, and fusion, while the semi-rigid segment of the Waveflex played a secondary role in the clinical symptoms of the patients, requiring partial decompression. In case of a certain degree of degeneration, the intervertebral disc with a Pfirrmann grade of at least 3 required semi-rigid internal fixation^18^.

In the PLIF group, according to the comprehensive judgment of symptoms, signs, and imaging, both segments were the significantly involved and played an important role in clinical symptoms. The degree of intervertebral disc degeneration was classified as Pfirrmann grade 3 or above^18^, and posterior decompression, fusion, and rigid fixation were needed.

### Evaluation indexes

Visual Analogue Scale (VAS) and Oswestry Disability Index (ODI) were used to evaluate clinical efficacy and Pfirrmann grade was used to evaluate the degree of intervertebral disc degeneration.

**Figure 2:**
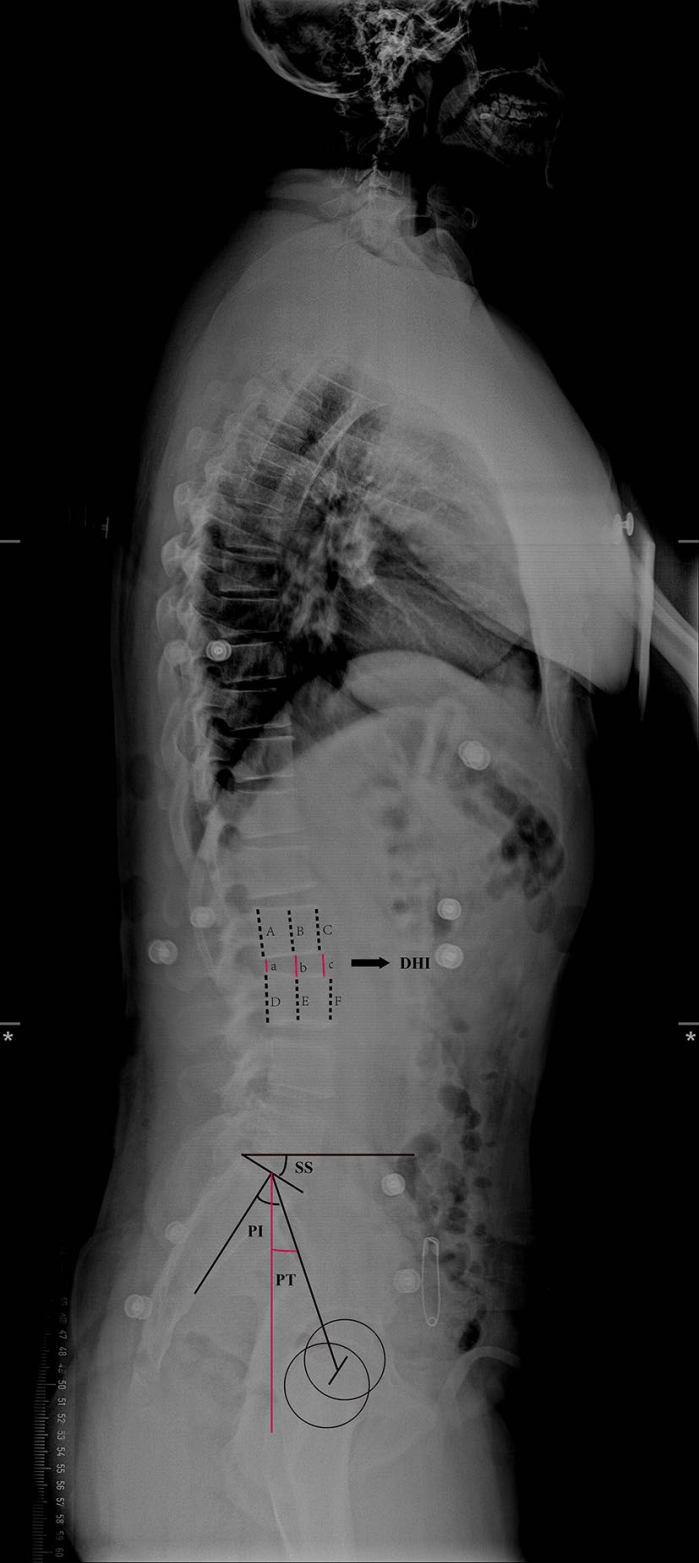
The definition of certain imaging parameters. Disc Height Index (DHI): The average of the anterior, middle, and posterior disc measurements was taken and it divided by the average of the heights of the adjacent upper and lower vertebral bodies, which were also the average of the heights of the anterior, middle, and posterior vertebral bodies. As shown in Fig. 1, DHI = (a + b + c)/(A + B + C + D + E + F)*2; Intervertebral Foramen Height (IFH): It was defined as the distance from the lowest point of the upper pedicle to the highest point of the lower pedicle on three-dimensional CT reconstruction; Range Of Motio (ROM): It was the angle that between the upper and lower endplate extension lines in the hyperextension position was subtracted from the angle between the upper and lower endplate extension lines in the hyperflexion position; Lumbar Lordosis (LL): It was the angle formed between the upper endplate of L1 and the upper endplate of S1; Pelvic Incidence (PI): It was defined as the angle between the line perpendicular to the sacral plate at its midpoint and the line connecting this point to the axis of the femoral heads; Sacral Slope (SS): It was defined as the angle between the superior plate of S1 and a horizontal line; Pelvic Tilt(PT): It was defined as the angle between the line connecting the midpoint of the sacral plate to the femoral heads axis and the vertical.

Imaging examinations were performed at different stages before and after surgery. Imaging parameters were obtained by X-ray for assessment of local disc degeneration of the cephalic adjacent segment (including disc height index (DHI), intervertebral foramen height (IFH), and ROM) and overall spinal motor function (including lumbar lordosis (LL), pelvic incidence (PI), sacral slope (SS), pelvic tilt (PT) and |PI-LL|). These parameters were measured independently by two observers and the final results were averaged. The definition of certain parameter is shown in Fig. 2.

### Statistical analysis

The statistical data were analysed by SPSS 25.0 statistical software (IBM SPSS Statistics for Windows, Version 25.0. Armonk, NY: IBM Corp), the measurement data were expressed by mean ± standard deviation, paired-samples *t* test was used for those with normal distribution and nonparametric rank-sum test was used for those without a normal distribution. Counting data were expressed in frequency or constituent ratio and were compared by the *χ*^2^ test. A *P* < 0.05 was considered statistically significant. The statistical analyses of this study were supervised and reviewed by statistical experts at Shandong University of Traditional Chinese Medicine.

## Results

### Baseline data

A total of 50 patients were enrolled in Waveflex (25 patients) and PLIF (25 patients) groups, with no cases dropped out. There were 8 male and 17 female patients in the Waveflex group (average follow-up time: 61.07 ± 0.63 months) and 10 male and 15 female patients in the PLIF group (average follow-up time: 61.04 ± 0.66 months). There were no significant differences between the two groups (*P* > 0.05) in the baseline variables, such as age, operation time, intraoperative blood loss, postoperative normal landing time, follow-up time, VAS score, ODI score and preoperative imaging parameters (Table 1).

**Table 1:** General baseline variables between Waveflex and PLIF group ($\overline{\user2{x}} \pm {\text{S}}$).

	Waveflex group	PLIF group	*P* value
Age (year)	67.04 ± 5.94	64.64 ± 13.51	0.420
Operation time (min)	109.28 ± 6.28	112.56 ± 6.93	0.086
Intraoperative blood loss (ml)	112.44 ± 8.69	115.44 ± 6.30	0.169
Postoperative normal landing time (day)	2.86 ± 0.73	3.12 ± 0.67	0.194
Follow-up time (month)	61.07 ± 0.63	61.04 ± 0.66	0.844
VAS score	7.00 ± 0.76	7.16 ± 0.80	0.284
ODI score	61.18% ± 5.05%	61.74% ± 4.84%	0.771
Preoperative imaging parameters			
DHI (%)	40.34% ± 1.90%	40.28% ± 1.90%	0.969
IFH (mm)	21.54 ± 1.08 mm	21.59 ± 0.94 mm	0.954
ROM (°)	8.68° ± 1.03°	8.86° ± 0.88°	0.299
LL (°)	41.80° ± 6.30°	42.08° ± 6.10°	0.839
PI (°)	55.74° ± 4.40°	55.89° ± 4.66°	0.900
|PI-LL (°)	13.94° ± 4.81°	13.81° ± 4.87°	0.900
PT (°)	20.88° ± 3.17°	21.06° ± 3.27°	0.712
SS (°)	34.86° ± 3.17°	34.83° ± 3.07°	0.999

### Clinical curative effect assessed by the VAS and ODI scores

Preoperative VAS and ODI scores were not significantly different between the Waveflex and PLIF groups (*P* > 0.05). Intra-group comparisons showed significant differences in VAS and ODI scores preoperatively and at follow-up (*P* < 0.05; Table 2).

**Table 2:** The between-group and intra-group comparison of VAS and ODI score between Waveflex and PLIF group at different time points ($\overline{\user2{x}} \pm {\text{S}}$).

	Preoperative data	Postoperative data at 3 months	Postoperative data at 1 year	Postoperative data at 5 year
VAS score				
Waveflex group	7.00 ± 0.76	3.20 ± 0.58	1.76 ± 0.52	0.68 ± 0.56
PLIF group	7.16 ± 0.80	3.32 ± 0.63	1.96 ± 0.45	0.56 ± 0.51
*Z* value	- 1.071	- 0.765	- 1.444	- 0.721
*P* value	0.284	0.444	0.149	0.471
ODI score				
Waveflex group	61.18% ± 5.05%	36.33% ± 3.50%	24.25% ± 2.69%	16.41% ± 1.86%
PLIF group	61.74% ± 4.84%	38.34% ± 3.29%	22.86% ± 2.31%	15.34% ± 2.38%
*Z* value	- 0.291	- 1.826	- 1.4	- 1.586
*P* value	0.771	0.068	0.162	0.113

	*Z* value		*P* value	
VAS score				
Waveflex group				
Postoperative data at 3 months. VS Preoperative data	− 6.252		0.000	
Postoperative data at 1 year VS Preoperative data	− 6.274		0.000	
Postoperative data at 5 years. VS Preoperative data	− 6.243		0.000	
PLIF group				
Postoperative data at 3 months. VS Preoperative data	− 6.205		0.000	
Postoperative data at 1 year VS Preoperative data	− 6.335		0.000	
Postoperative data at 5 years. VS Preoperative data	− 6.228		0.000	
ODI score				
Waveflex group				
Postoperative data at 3 months. VS Preoperative data	− 6.065		0.000	
Postoperative data at 1 year VS Preoperative data	− 6.067		0.000	
Postoperative data at 5 years. VS preoperative data	− 6.068		0.000	
PLIF group				
Postoperative data at 3 months. VS preoperative data	− 6.070		0.000	
Postoperative data at 1 year VS preoperative data	− 6.066		0.000	
Postoperative data at 5 years. VS preoperative data	− 6.067		0.000	

### Imaging indexes assessment

#### Local intervertebral disc degeneration evaluation

**Figure 3:**
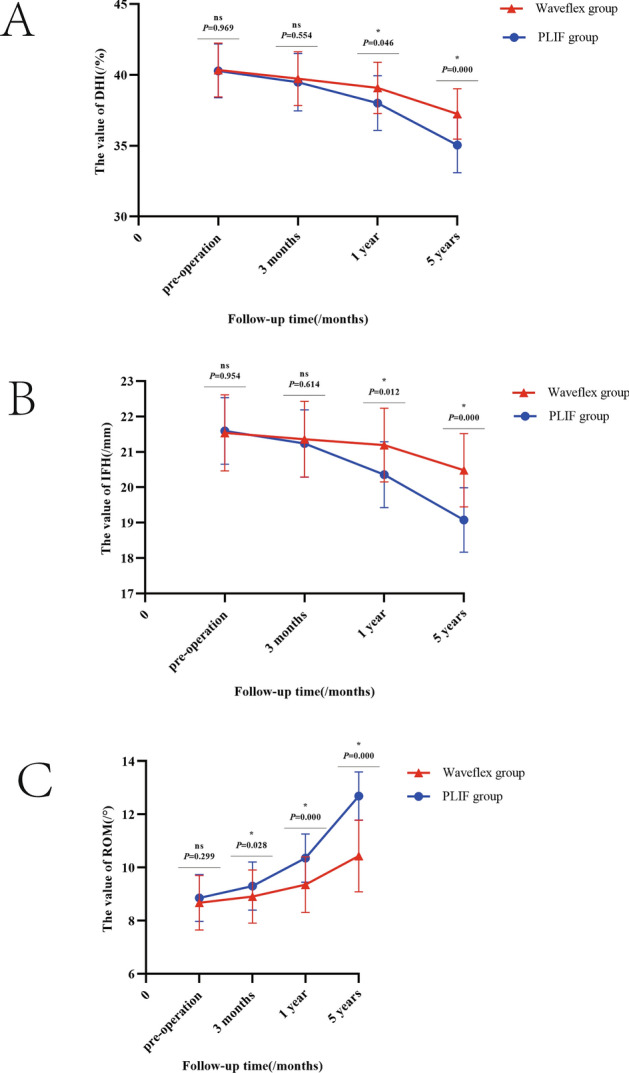
(**A**) Graph of the comparison in DHI value between Waveflex and PLIF group. (**B**) Graph of the comparison in IFH value between Waveflex and PLIF group. (**C**) Graph of the comparison in ROM value between Waveflex and PLIF group.

There were no significant differences in the DHI and IFH values between the Waveflex and PLIF groups preoperatively and at 3 months postoperatively (*P* > 0.05), but there were significant differences at 1 and 5 years after surgery (*P* < 0.05) (see Fig. 3A,B). There was no significant difference in ROM between the two groups before operation (*P* > 0.05), but there were significant differences in ROM values at 3 months, 1 year, and 5 years postoperatively (*P* < 0.05) (see Fig. 3C). The intra-group comparison showed significant differences in both groups for the DHI, IFH, and ROM values at 5 year postoperatively, when compared with the pre-operative values (*P* < 0.05; Table 3).

**Table 3:** The between-group and intra-group of DHI, IFH, ROM values between Waveflex and PLIF group at different time points ($\overline{\user2{x}} \pm {\text{S}}$).

	Preoperative data	Postoperative data at 3 months	Postoperative data at 1 year	Postoperative data at 5 year
DHI				
Waveflex group	40.34% ± 1.90%	39.73% ± 1.90%	39.08% ± 1.82%	37.24% ± 1.78%
PLIF group	40.28% ± 1.90%	39.48% ± 2.03%	38.00% ± 1.93%	35.04% ± 1.95%
*Z* value	− 0.039	− 0.592	− 1.998	− 3.719
*P* value	0.969	0.554	0.046	0.000
IFH				
Waveflex group	21.54 ± 1.08 mm	21.36 ± 1.07 mm	21.19 ± 1.04 mm	20.48 ± 1.04 mm
PLIF group	21.59 ± 0.94 mm	21.24 ± 0.95 mm	20.36 ± 0.93 mm	19.08 ± 0.91 mm
*Z* value	− 0.058	− 0.505	− 2.513	− 4.124
*P* value	0.954	0.614	0.012	0.000
ROM				
Waveflex group	8.68° ± 1.03°	8.91° ± 1.00°	9.36° ± 1.05°	10.44° ± 1.35°
PLIF group	8.86° ± 0.88°	9.30° ± 0.91°	10.36° ± 0.91°	12.69° ± 0.91°
*Z* value	− 1.038	− 2.203	− 3.950	− 5.318
*P* value	0.299	0.028	0.000	0.000

	*Z* value		*P* value	
DHI				
Waveflex group				
Postoperative data at 3 months. VS Preoperative data	− 1.126		0.260	
Postoperative data at 1 year VS Preoperative data	− 2.171		0.030	
Postoperative data at 5 years. VS Preoperative data	− 4.558		0.000	
PLIF group				
Postoperative data at 3 months. VS Preoperative data	− 1.572		0.116	
Postoperative data at 1 year VS Preoperative data	− 3.782		0.000	
Postoperative data at 5 years. VS Preoperative data	− 5.720		0.000	
IFH				
Waveflex group				
Postoperative data at 3 months. VS Preoperative data	− 0.805		0.421	
Postoperative data at 1 year VS Preoperative data	− 1.281		0.200	
Postoperative data at 5 years. VS Preoperative data	− 3.153		0.020	
PLIF group				
Postoperative data at 3 months. VS Preoperative data	− 1.475		0.140	
Postoperative data at 1 year VS Preoperative data	− 4.017		0.000	
Postoperative data at 5 years. VS Preoperative data	− 5.744		0.000	
ROM				
Waveflex group				
Postoperative data at 3 months. VS Preoperative data	− 1.281		0.200	
Postoperative data at 1 year VS Preoperative data	− 3.436		0.001	
Postoperative data at 5 years. VS Preoperative data	− 4.376		0.000	
PLIF group				
Postoperative data at 3 months. VS Preoperative data	− 2.562		0.010	
Postoperative data at 1 year VS Preoperative data	− 4.619		0.000	
Postoperative data at 5 years. VS Preoperative data	− 6.065		0.000	

#### Overall spinal motor function evaluation

**Figure 4:**
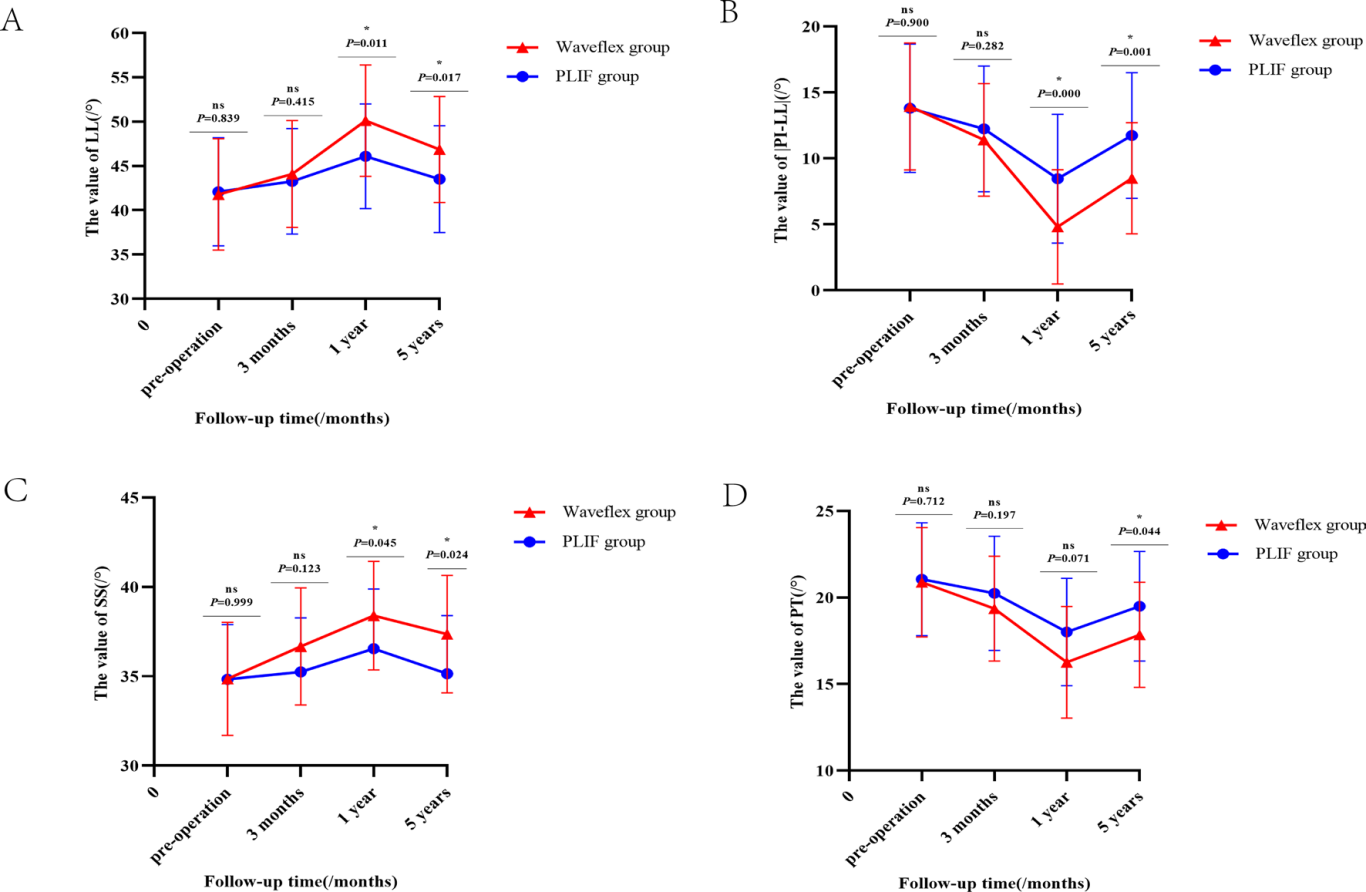
(**A**) Graph of the comparison in LL value between Waveflex and PLIF group. (**B**) Graph of the comparison in |PI-LL| value between Waveflex and PLIF group. (**C**) Graph of the comparison in SS value between Waveflex and PLIF group. (**D**) Graph of the comparison in PT value between Waveflex and PLIF group.

There were no significant differences in LL, |PI-LL|, and SS values between the Waveflex and PLIF groups preoperatively and at 3 months postoperatively (*P* > 0.05). However, there were significant differences between the two groups at 1 and 5 years postoperatively (*P* < 0.05) (see Fig. 4A–C). There was no significant difference in PT values between the two groups preoperatively, 3 months, and 1 year postoperatively (*P* > 0.05), but the PT values were significantly different 5 years postoperatively (*P* < 0.05; see Fig. 4D). The intra-group comparison of the Waveflex group showed significant improvements in |PI-LL| and PT values (*P* < 0.05) when compared to those of the preoperative stage. In the PLIF group, there was a significant difference in |PI-LL| and PT values at 1 and 5 years after operation compared with those before operation (*P* < 0.05). LL and SS values of the Waveflex group were significantly improved 1 and 5 years postoperatively (*P* < 0.05), while there was no significant improvement in the PLIF group at follow-up (*P* > 0.05). Conversely, PI values were not significantly different either between the groups or in the intra-group comparison at any time point (*P* > 0.05; Table 4). We also provided two complete typical cases as supplemental digital content: Typical case data 1 and 2 (Figs. 5–11).

**Table 4:** The between-group and intra-group of LL, PI, |PI-LL|, PT, SS values between Waveflex and PLIF group at different time points ($\overline{\user2{x}} \pm {\text{S}}$).

	Preoperative data	Postoperative data at 3 months	Postoperative data at 1 year	Postoperative data at 5 year
LL				
Waveflex group	41.80° ± 6.30°	44.08° ± 6.03°	50.10° ± 6.29°	46.86° ± 5.97°
PLIF group	42.08° ± 6.10°	43.26° ± 5.96°	46.08° ± 5.90°	43.50° ± 6.04°
*Z* value	− 0.204	− 0.815	− 2.532	− 2.396
*P* value	0.839	0.415	0.011	0.017
PI				
Waveflex group	55.74 ± 4.40°	55.47 ± 4.46°	54.92 ± 4.32°	55.35 ± 4.36°
PLIF group	55.89 ± 4.66°	55.48 ± 4.66°	54.54 ± 4.74°	55.23 ± 4.68°
*Z* value	− 0.126	− 0.049	− 2.081	− 0.126
*P* value	0.900	0.961	0.778	0.900
|PI-LL|				
Waveflex group	13.94 ± 4.81°	11.40 ± 4.26°	4.82 ± 4.33°	8.49 ± 4.21°
PLIF group	13.81 ± 4.87°	12.23 ± 4.76°	8.46 ± 4.88°	11.74 ± 4.76°
*Z* value	− 0.126	− 1.077	− 3.522	− 3.463
*P* value	0.900	0.282	0.000	0.001
PT				
Waveflex group	20.88 ± 3.17°	19.35 ± 3.03°	16.25 ± 3.23°	17.84 ± 3.04°
PLIF group	21.06 ± 3.27°	20.24 ± 3.30°	18.00 ± 3.11°	19.49 ± 3.17°
*Z* value	− 0.369	− 1.291	− 1.805	− 2.018
*P* value	0.712	0.197	0.071	0.044
SS				
Waveflex group	34.86 ± 3.17°	36.67 ± 3.27°	38.40 ± 3.04°	37.35 ± 3.29°
PLIF group	34.83 ± 3.07°	35.24 ± 3.03°	36.54 ± 3.34°	35.15 ± 3.24°
*Z* value	0	− 1.543	− 2.008	− 2.251
*P* value	0.999	0.123	0.045	0.024

	*Z* value		*P* value	
LL				
Waveflex group				
Postoperative data at 3 months. VS Preoperative data	− 1.785		0.074	
Postoperative data at 1 year VS Preoperative data	− 3.949		0.000	
Postoperative data at 5 years. VS Preoperative data	− 2.920		0.003	
PLIF group				
Postoperative data at 3 months. VS Preoperative data	− 1.106		0.269	
Postoperative data at 1 year VS Preoperative data	− 2.455		0.014	
Postoperative data at 5 years. VS Preoperative data	− 1.271		0.204	
PI				
Waveflex group				
Postoperative data at 3 months. VS Preoperative data	− 0.417		0.677	
Postoperative data at 1 year VS Preoperative data	− 0.844		0.399	
Postoperative data at 5 years. VS Preoperative data	− 0.470		0.635	
PLIF group				
Postoperative data at 3 months. VS Preoperative data	− 0.437		0.662	
Postoperative data at 1 year VS Preoperative data	− 0.912		0.362	
Postoperative data at 5 years. VS Preoperative data	− 0.553		0.580	
|PI-LL|				
Waveflex group				
Postoperative data at 3 months. VS Preoperative data	− 2.784		0.005	
Postoperative data at 1 year VS Preoperative data	− 5.142		0.000	
Postoperative data at 5 years. VS Preoperative data	− 4.288		0.000	
PLIF group				
Postoperative data at 3 months. VS Preoperative data	− 1.659		0.097	
Postoperative data at 1 year VS Preoperative data	− 4.026		0.000	
Postoperative data at 5 years. VS Preoperative data	− 2.008		0.045	
PT				
Waveflex group				
Postoperative data at 3 months. VS Preoperative data	− 2.232		0.026	
Postoperative data at 1 year VS Preoperative data	− 4.521		0.000	
Postoperative data at 5 years. VS Preoperative data	− 3.405		0.001	
PLIF group				
Postoperative data at 3 months. VS Preoperative data	− 1.145		0.252	
Postoperative data at 1 year VS Preoperative data	− 3.493		0.000	
Postoperative data at 5 years. VS Preoperative data	− 2.086		0.037	
SS				
Waveflex group				
Postoperative data at 3 months. VS Preoperative data	− 2.028		0.200	
Postoperative data at 1 year VS Preoperative data	− 3.366		0.001	
Postoperative data at 5 years. VS Preoperative data	− 2.561		0.010	
PLIF group				
Postoperative data at 3 months. VS Preoperative data	− 0.728		0.467	
Postoperative data at 1 year VS Preoperative data	− 1.834		0.667	
Postoperative data at 5 years. VS Preoperative data	− 0.524		0.600	

## Discussion

Previous studies have shown that, compared with the rigid internal fixation of interbody fusion, the dynamic pedicle fixation system significantly reduces the influence of surgery on the original biomechanical basis of the spine, maintaining, as far as possible, the uniform distribution and normal function of the motion load of each intervertebral disc of the spine^19^. This fixation method is widely used in the clinic, as it preserves the segmental movement of the surgical site and effectively relieves the degeneration of adjacent segments^20^. However, most of these studies were short-term and with a focus on local recovery of the surgical site, thus ignoring the surgery effect on spinal motor function. Nonetheless, the spine should be viewed as a whole. Achieving the best spinal alignment and ensuring sagittal balance are important factors for postoperative recovery and long-term spinal stability^21^. Therefore, our study comprehensively and systematically investigated the clinical efficacy and imaging parameters of the Waveflex semi-rigid-dynamic-internal-fixation system for the treatment of lumbar degenerative diseases from two perspectives: local and overall. As far as we know, ours is the first study to prove the Waveflex semi-rigid-dynamic-internal-fixation system can not only delay postoperative adjacent intervertebral disc segment degeneration, but also improve lumbar kyphosis and sagittal imbalance caused by long-term recurrent lumbar degenerative diseases. Furthermore, it is also a highly safe treatment, with long-term efficacy.

Here, the VAS and ODI scores of patients in the Waveflex group were significantly improved 1 and 5 years postoperatively (*P* < 0.05), suggesting the obvious clinical effect of the approach. Furthermore, the clinical symptoms were further improved within 1 year after operation and subsequently stabilized, with no further improvement, recurrence, or aggravation during the 5-year follow-up.

In the local intervertebral disc degeneration assessment, the Waveflex group showed obvious symptom improvement and clinical efficacy compared with the PLIF group. Related biomechanical studies have pointed out that the significant decrease in the ROM of the fusion segments leads to excessive stress concentration in the adjacent segments^22^. Moreover, the compensatory ROM increase, resulting in the change of the original sagittal balance and biomechanics, leads to the degeneration of the adjacent segments and loss of intervertebral space and IFHs, which are the main manifestations of degeneration^23^. Several studies have shown that, compared with the rigid fixation system, the pedicle dynamic fixation system can maintain the ROM of the operative segment, significantly shorten operation time, and reduce blood loss, hospital stay, and postoperative complications^24^. Especially in the medium- and long-term follow-ups, and unlike the high incidence of adjacent segment degeneration caused by rigid lumbar fixation, pedicle dynamic fixation can maintain partial segmental motion and intervertebral height, while delaying the progression of adjacent segment degeneration^25,26^, which is consistent with our results. Although patients in the Waveflex and PLIF groups had different degrees of adjacent segment degeneration postoperatively, the degeneration process in the Waveflex group was significantly slower than that of the PLIF group.

At 1-year and 5-years follow-up, the DHI and IFH values of the adjacent segment cephalic side from the two groups present the decline of different degree, but at 5-years follow-up, the PLIF group decreased more rapidly, and the difference between the two groups was statistically significant (*P* < 0.05). This shows that the semi-rigid internal fixation system can effectively maintain the height of adjacent segmental intervertebral space, which may be related to the support of semi-rigid titanium rods. We believe that the elastic fixation of the semi-rigid internal fixation system can effectively alleviate the mechanical conduction between the surgical segment and the adjacent segment, effectively alleviating the load distribution of the adjacent intervertebral disc. Concomitantly, the pre-bent structure of the semi-rigid titanium rod also has a positive impact on the recovery of the physiological curvature of lumbar vertebrae. The ROM of the adjacent segment showed no significant differences between the two groups before operation (*P* > 0.05), but the ROM of the adjacent segment increased in varying degrees postoperatively. During the 3-month follow-up, there were significant differences in ROM values between the Waveflex and PLIF groups, indicating that semi-rigid fixation has obvious clinical effects during early postoperative stages, and can effectively alleviate the early excessive activity of adjacent segments. At the 5-year follow-up, the ROM value increased from 8.68° ± 1.03° to 10.44° ± 1.35° in the Waveflex group and from 8.86° ± 0.88° to 12.69° ± 0.91° in the PLIF group. In conclusion, the Waveflex semi-rigid-dynamic-internal-fixation system was able to delay the excessive ROM increase in the adjacent segment and degeneration of the intervertebral disc to a certain extent in terms of long-term efficacy.

Regarding overall spinal motor function evaluation, the Waveflex group showed obvious advantages and clinical efficacy compared with the PLIF group. Lumbar degenerative disease leads to decrease of intervertebral space height, increase of corresponding segmental ROM, and change of lumbar biomechanical properties, which reduces LL and SS and increases PI. The changes in these parameters will significantly affect the stress distribution on the lumbar spine, leading to excessive fatigue of the lower back muscles, thus aggravating the degeneration of the intervertebral discs^27^. A large number of studies^28–31^ have pointed out that the quality of life score (SF-36 score) decreased in patients with loss of lumbar kyphosis angle, however, effective and sufficient restoration of LL can substantially improve the patient's dysfunction, reduce sagittal decompensation, and reduce complications after fusion. Suzuki et al.^32^ found that patients with lumbar spinal stenosis and intermittent claudication relieve back and lower limb numbness pain by forward flexion while walking, but long-term flexion in daily life makes lumbar kyphosis difficult to maintain due to decreased strength of the paraspinal muscles and degenerative atrophy, which leads to a vicious circle of abnormal posture. Simultaneously, the results also showed that a smaller LL angle and a larger pelvic incidence angle may make intermittent claudication more likely^32^. This further indicates that the sagittal sequence of the spine was worse in patients with more severe pelvic retroversion and trunk flexion. When exploring the relationship between SS, LL, and lower back pain, Liow et al. found that, compared with patients with lumbar spondylolisthesis and postoperative SS ≤ 30°, patients with increased SS at the last follow-up (SS ≥ 30°) had less lower back pain at 6 months and 2 years after short-segment lumbar fusion, resulting in a better sagittal balance postoperatively, which was related to larger LL postoperatively^33^. Our study shows that, although there were varying degrees of improvement in overall spinal motor function of patients in both the Waveflex and the PLIF groups, the improvement in the Waveflex group is stronger than that in the PLIF group. LL, PI, |PI-LL|, PT and SS were included in the statistics. Except for PI, the other four variables had statistically significant differences between baseline and 1-year postoperatively (*P* < 0.05). However, during the 1-year follow-up, the value of LL, |PI-LL|, PT and SS from the two groupsshowed changes to varying degrees. There were significant differences between the two groups (*P* < 0.05). These results suggest that both the Waveflex semi-rigid-dynamic-internal-fixation system and posterior lumbar interbody fusion can improve the lumbar kyphosis angle and sagittal position of spine in a short time, correct the preoperative spinopelvic mismatch, and improve the quality of life of patients postoperatively. However, the data of each group had a change in varying degrees at the 5-year follow-up compared with the 1-year follow-up. This may be due to the effective interbody fusion and recovery of soft tissue postoperatively, which effectively alleviated the damage to the posterior ligament complex during the operation and ameliorated the adverse effects of internal fixation on the sagittal balance of the lumbar spine. Nonetheless, spinal biomechanical changes and degeneration of the intervertebral disc brought by long-term lumbar fusion will lead to a new spinal-balance state. This balance is still improved when compared with that before surgery. Concurrently, during the 5-year follow-up, the degeneration degree of spinopelvic characteristics in the Waveflex group was significantly lower than that in the PLIF group, indicating that the Waveflex semi-rigid fixation is better than rigid fixation for improving spinal sagittal imbalance and delaying the degenerative changes of the lumbar spine.

Although this study provides some meaningful evidence for clinical practice, it has several limitations. First, due to the limited number of cases in this single-centre study, wider and multi-centre studies are needed to provide higher levels of evidence and verify our results. Second, the patients were not randomly assigned, and they were grouped according to the surgical procedure chosen by themselves, which may lead to selection bias. Finally, although no postoperative complications were reported in our study, there is still a certain risk in long-term use of elastic titanium rods. Therefore, more in-depth and long-term research on this system in the future is needed to explore its advantages and disadvantages.

## Conclusion

The Waveflex semi-rigid dynamic fixation system is a long-term safe and effective surgical method and, compared with rigid internal fixation with interbody fusion, it can effectively reduce the probability of intervertebral disc degeneration in superior adjacent segments. At the same time, it can improve the LL angle and spinal sagittal imbalance, while improving the quality of life of patients postoperatively.

### Supplementary Information

Supplementary Information.

## Data Availability

All data generated or analysed during this study are included in this published article.
