# Supplementary material for: Long-term efficacy of Waveflex semi-rigid-dynamic-internal-fixation system in delaying intervertebral disc degeneration at adjacent segments and improving spinal sagittal imbalance
Source: Sci Rep. 2024 May 7;14:10437. doi: 10.1038/s41598-024-60940-8 (PMC11076526; doi:10.1038/s41598-024-60940-8)
Supplement: Supplementary file 1 — Supplementary Information. [file 41598_2024_60940_MOESM1_ESM.docx]

Typical case data 1:

A 57-year-old female patient complained of low back pain for 8 months, which worsened with pain in the left lower limb for 50 days. Physical examination showed spinous process and paraspinal tenderness (+) and percussion pain (+) in L4, L5, S1, with radiating pain in the left lower limb. Superficial skin sensation of the lateral calf, dorsal foot and lateral plantar skin of the left lower limb was decreased, while the superficial skin sensation of the right lower limb was normal, and the key muscle strength of lower limb was normal. The patient was diagnosed with lumbar spinal stenosis (L4/5, L5/S1). The surgical plan was L4/5 Waveflex semi-rigid fixation + left hemilaminectomy decompression and interbody fusion with internal fixation at L5/S1.

As can be seen from MRI, the patient's intervertebral discs in L4/5 and L5/S1 appeared more serious spinal stenosis, the lumbar physiological curvature became straight, and other lumbar segments appeared mild intervertebral disc degeneration. In order to slow down the patient's lumbar spine overall degeneration, and restore a certain physiological curvature through surgery, we chose this surgical method. Through the follow-up found that, It is true that the preoperative goal is achieved through surgery.

Figure5
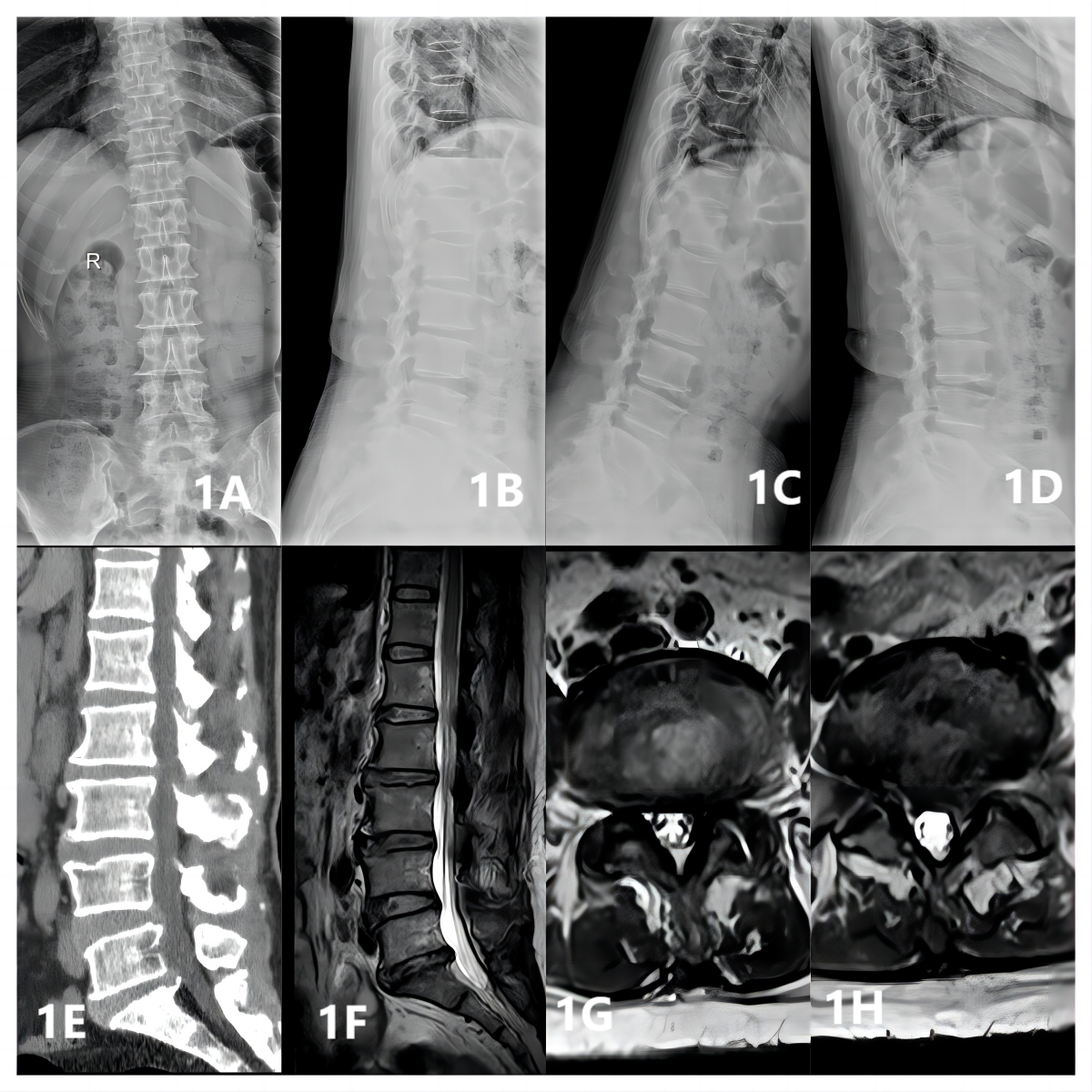


1A-1H was the preoperative imaging data of the patient, which showed that the patient's spinal physiological curvature was straight, L5/S1 disc protrusion and calcification. 1G was the L4/5 disc MRI plain scan image, 1H was the L5/S1 disc MRI plain scan image.

Figure6
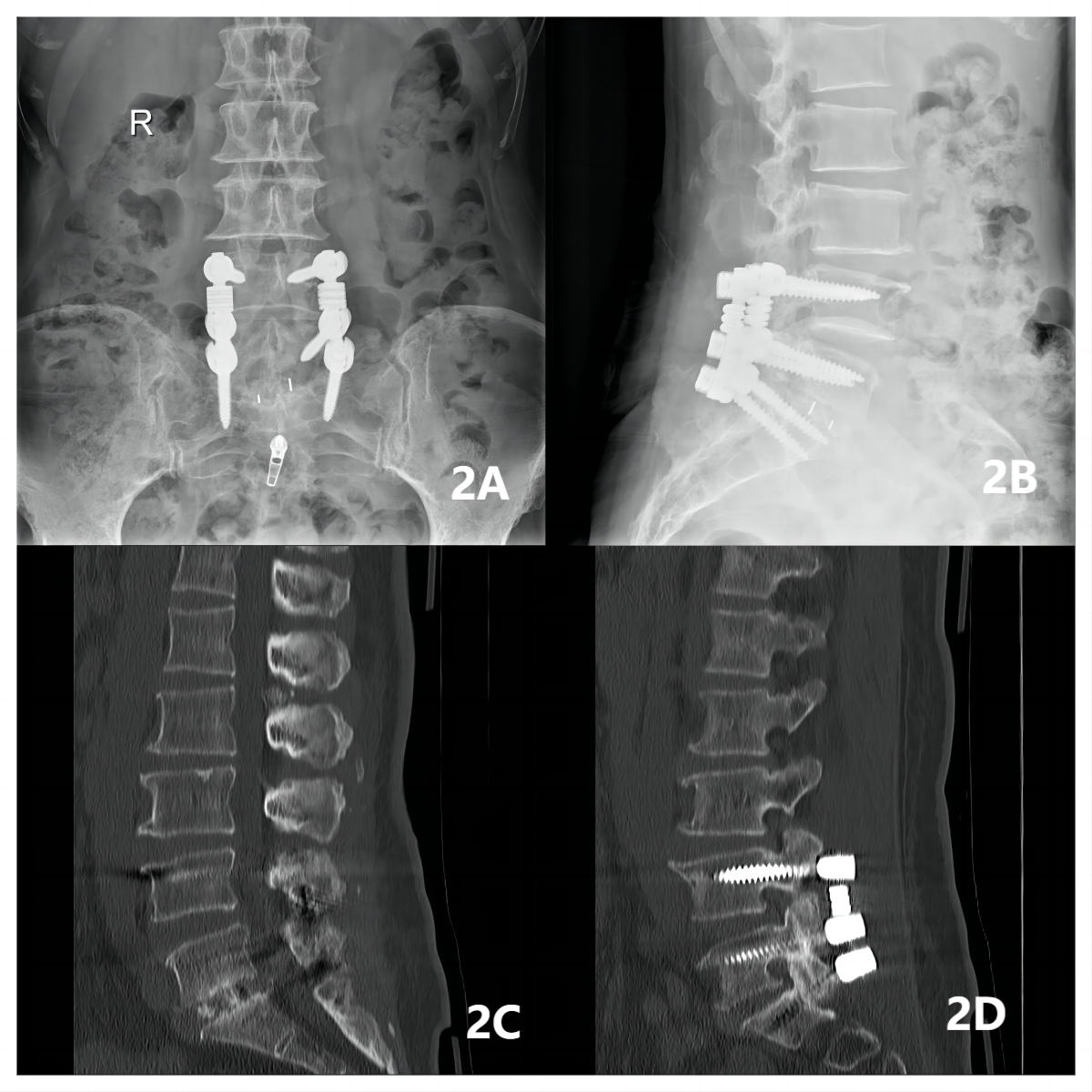


2A and 2B were the imaging data of patients at 3 months after operation. The imaging data of 2C and 2D at 1 year after operation showed that the intervertebral fusion was good and the position of the screw rod was satisfactory.

Figure7
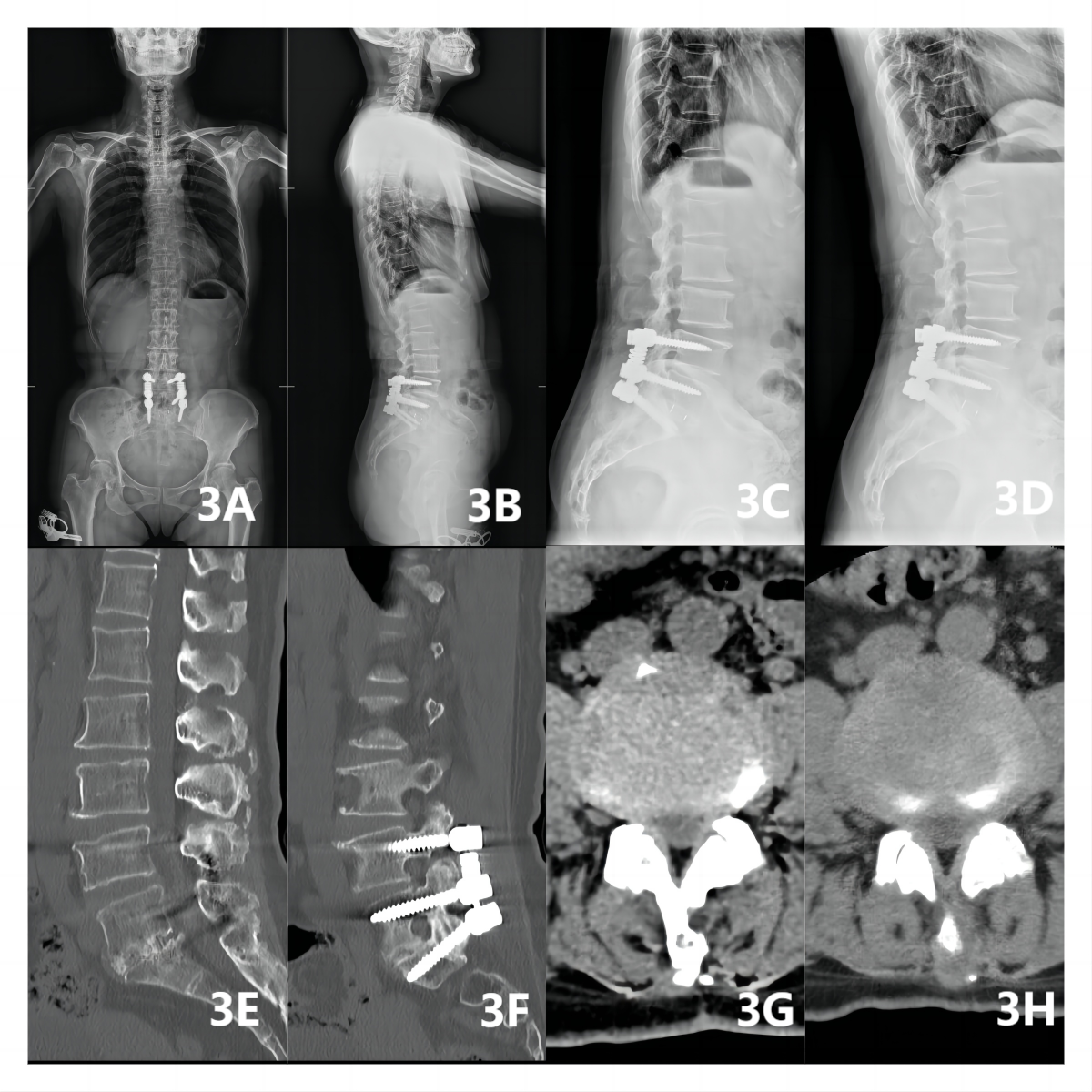


3A-3H was the imaging data of the patient at the 5-year follow-up after surgery. The physiological curvature of the spine was recovered when compared with that before operation, the position of the screw rod was good, and the L3/4 intervertebral space height and dynamic position range of motion had no significant change. 3G was the L3/4 intervertebral disc CT scan image 5 years after operation, 3H was the L3/4 intervertebral disc CT scan image before operation. There was no significant difference between the two groups.

Typical case data 2:

A 72-year-old man complained of pain and numbness in the right lower limb for 25 years and intermittent claudication for more than 3 years, which was aggravated for 2 days. Physical examination showed spinous process and paraspinal tenderness (+) and percussion pain (+) in L4. Superficial skin sensation in the right thigh and posterolateral skin of the right leg was decreased and lumbar dorsiflexion test was positive, while the superficial skin sensation of the left lower limb was normal, and the key muscle strength of lower limb was normal. The patient was diagnosed with lumbar spondylolisthesis (L4, degenerative) and lumbar disc herniation (L3/4). The surgical plan was L3/4 Waveflex semi-rigid fixation + Total laminectomy decompression and interbody fusion with internal fixation at L4/5.

As can be seen from MRI, the patient's intervertebral discs in L3/4 and L4/5 appeared more serious spinal stenosis, L4 vertebral body appeared spondylolisthesis. L2/3 segments appeared intervertebral disc degeneration, however, there were no corresponding clinical symptoms. In order to retain the patient's lumbar spine mobility as much as possible, and to solve the current clinical problems, we chose this surgical method, and through the follow-up found that, It is true that the preoperative goal is achieved through surgery.

Figure8
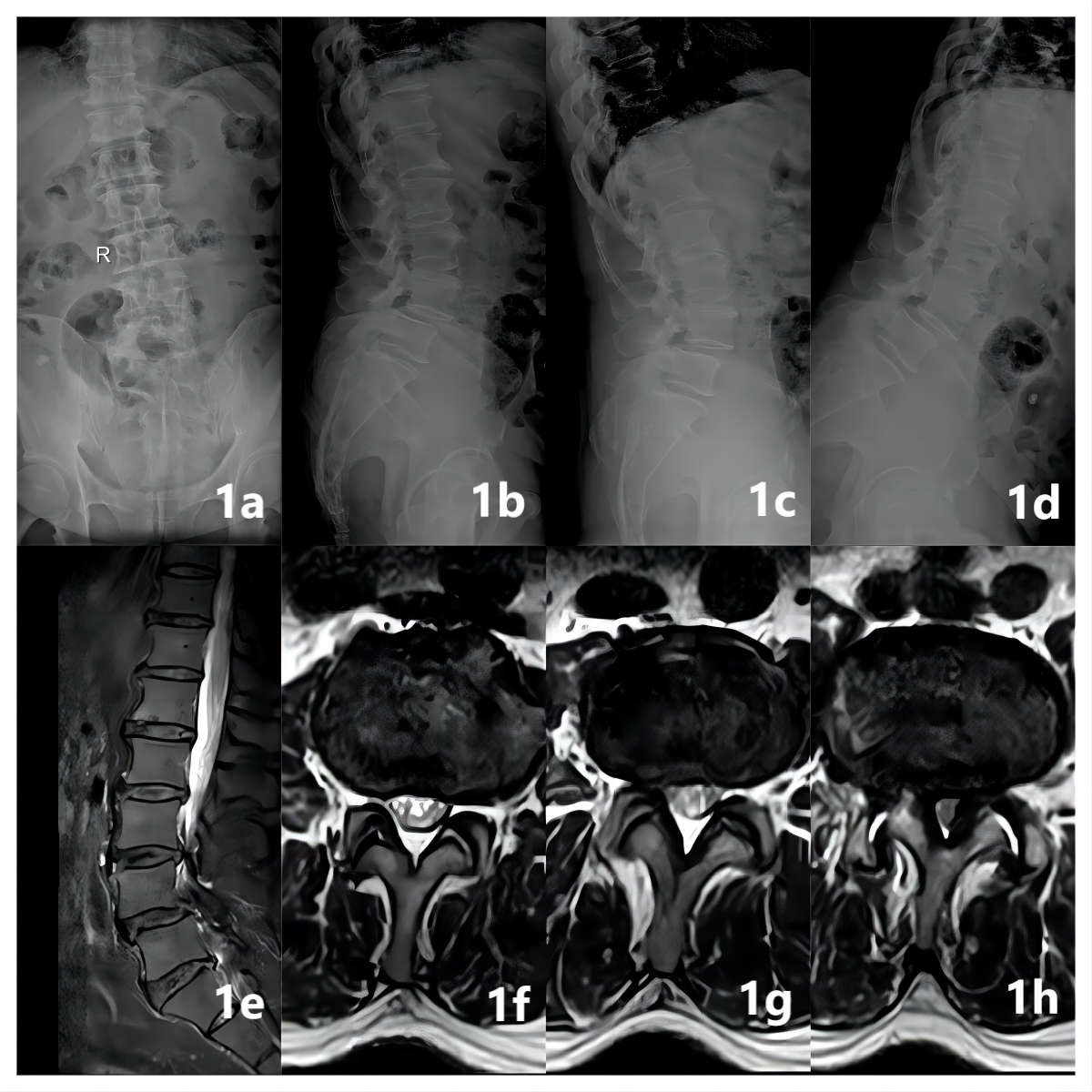


1a-1h was the preoperative imaging data of the patient, which showed that the L4 vertebral body of the patient was slightly spondylolisthesis forward, the L4/5 intervertebral disc level spinal canal and bilateral nerve root canal were narrowed, and the L3/4 intervertebral disc was herniated backward, compressing the dural sac. 1f was the L2/3 intervertebral disc MRI plain scan image, 1g was the L3/4 intervertebral disc MRI plain scan image, 1h was the L4/5 intervertebral disc MRI plain scan image.

Figure9
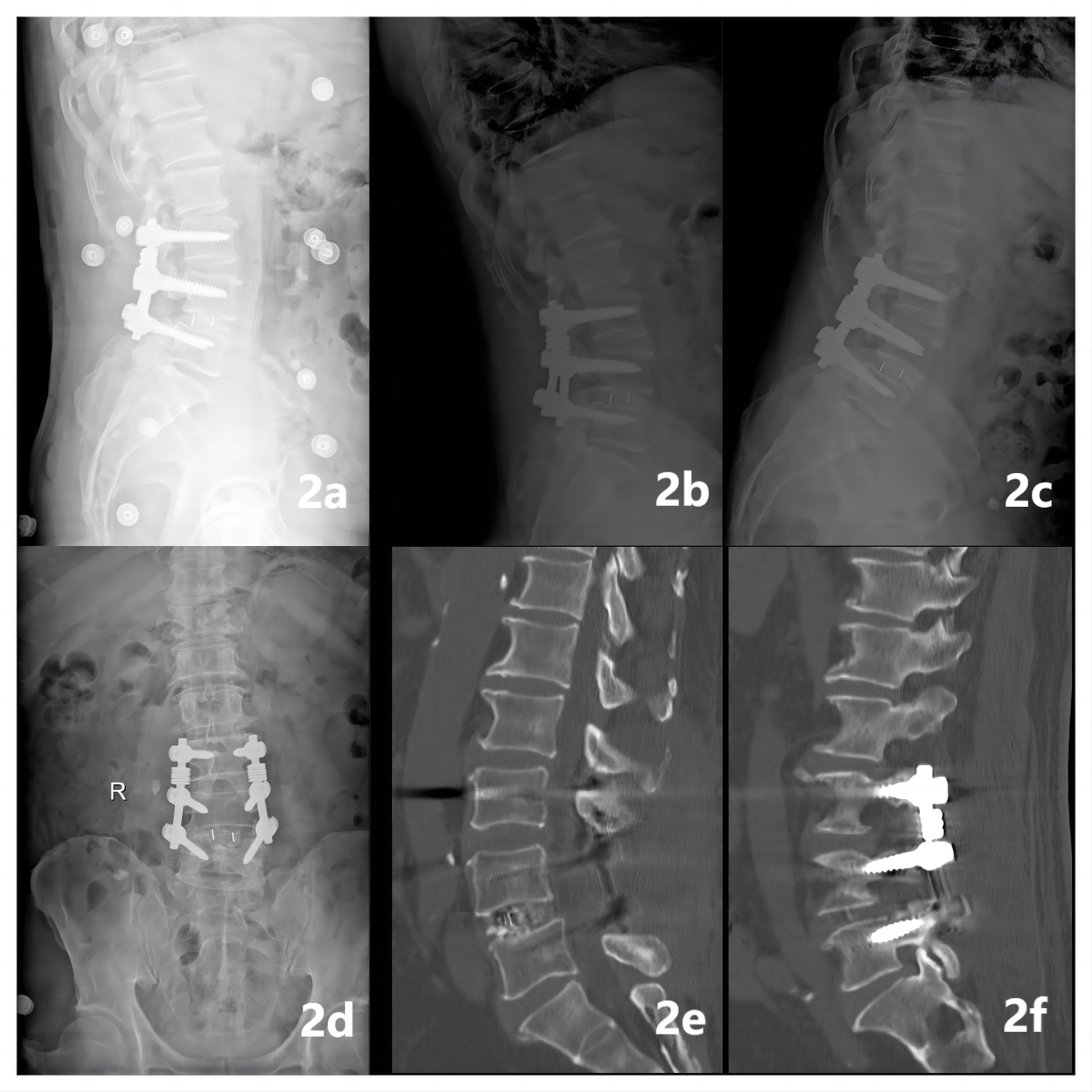


2a-2f was the imaging data of patients at 3 months after surgery which showed good reduction of the vertebral body and satisfactory position of the screw rod.

Figure10
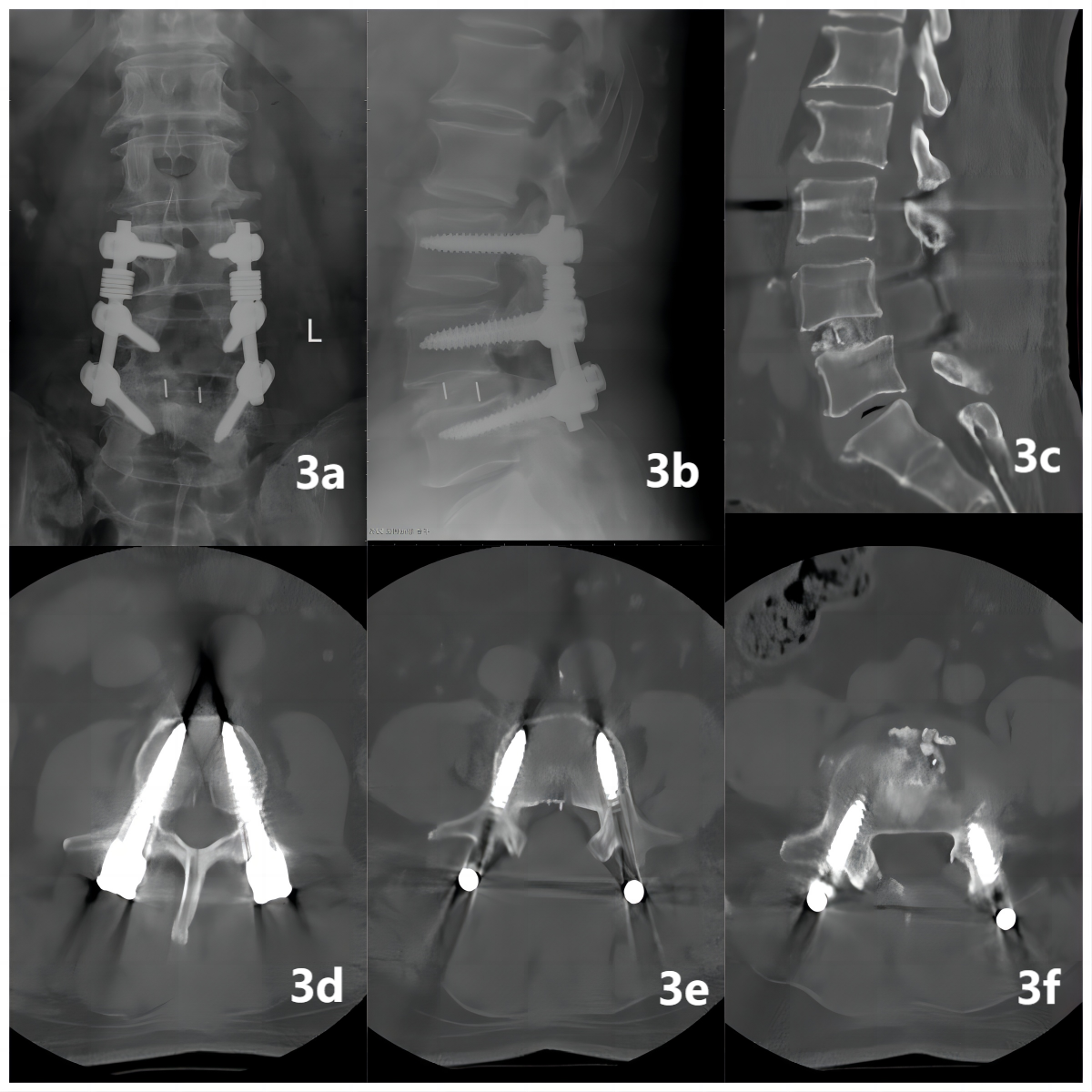


3a-3f was the imaging data of patients at 1 year after surgery which showed that the vertebral body was relatively stable, the interbody fusion was good, and the screw rod was not abnormal loose.

Figure11
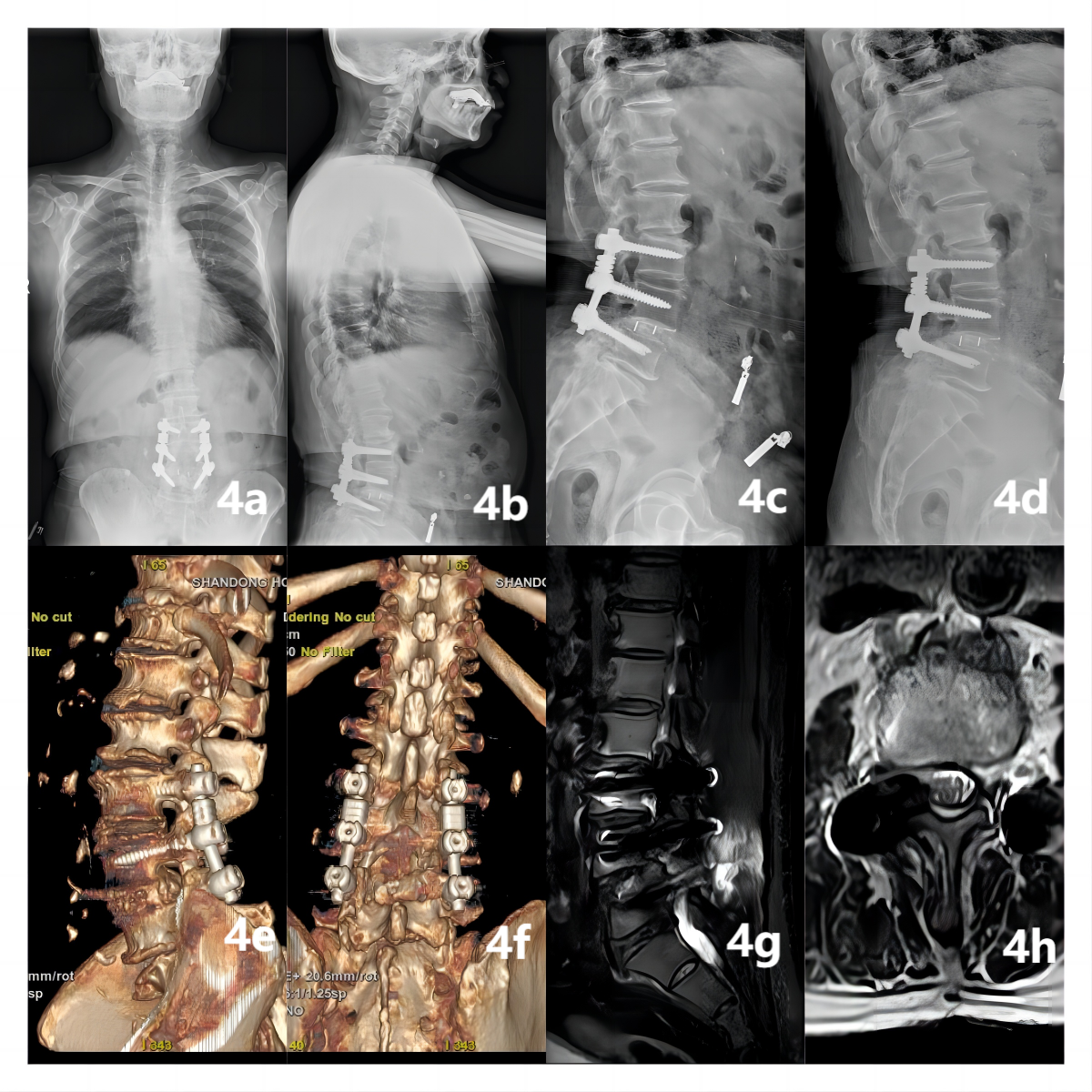


4a-4h was the imaging data of the patient at the 5-year follow-up after surgery. The physiological curvature of lumbar spine was restored compared with that before operation. The spinal sequence was stable and the position of the screw rod was good. The height of the L2/3 intervertebral space was slightly decreased compared with that before, and the dynamic position of the ROM had no significant change. 4h was the L2/3 intervertebral disc MRI plain scan image 5 years after surgery, and there was no significant change compared with that before.
